# Lysophosphatidic acid-3 receptor-mediated feed-forward production of lysophosphatidic acid: an initiator of nerve injury-induced neuropathic pain

**DOI:** 10.1186/1744-8069-5-64

**Published:** 2009-11-13

**Authors:** Lin Ma, Hitoshi Uchida, Jun Nagai, Makoto Inoue, Jerold Chun, Junken Aoki, Hiroshi Ueda

**Affiliations:** 1 Division of Molecular Pharmacology and Neuroscience, Nagasaki University Graduate School of Biomedical Sciences, 1-14 Bunkyo-machi, Nagasaki 852-8521, Japan; 2 Department of Molecular Biology, Helen L. Dorris Child and Adolescent Neuropsychiatric Disorder Institute, The Scripps Research Institute, 10550 North Torrey Pines Road, ICND118, La Jolla, CA 92037, USA; 3 Laboratory of Molecular and Cellular Biochemistry, Graduate School of Pharmaceutical Sciences, Tohoku University, Sendai, Japan

## Abstract

**Background:**

We previously reported that intrathecal injection of lysophosphatidylcholine (LPC) induced neuropathic pain through activation of the lysophosphatidic acid (LPA)-1 receptor, possibly via conversion to LPA by autotaxin (ATX).

**Results:**

We examined *in vivo *LPA-induced LPA production using a biological titration assay with B103 cells expressing LPA_1 _receptors. Intrathecal administration of LPC caused time-related production of LPA in the spinal dorsal horn and dorsal roots, but not in the dorsal root ganglion, spinal nerve or sciatic nerve. LPC-induced LPA production was markedly diminished in ATX heterozygotes, and was abolished in mice that were deficient in LPA_3_, but not LPA_1 _or LPA_2 _receptors. Similar time-related and LPA_3 _receptor-mediated production of LPA was observed following intrathecal administration of LPA. In an *in vitro *study using spinal cord slices, LPA-induced LPA production was also mediated by ATX and the LPA_3 _receptor. Intrathecal administration of LPA, in contrast, induced neuropathic pain, which was abolished in mice deficient in LPA_1 _or LPA_3 _receptors.

**Conclusion:**

These findings suggest that feed-forward LPA production is involved in LPA-induced neuropathic pain.

## Background

Lysophosphatidylcholine (LPC, lysolecithin) is the most abundant lysophospholipid in the blood and tissues [1,2]. LPC, an important biologically active signaling molecule, is generated under specific physiological and pathological conditions, and exerts multiple effects in atherosclerosis and inflammatory diseases [3-9]. LPC is widely reported to possess demyelinating properties, and has been used to study the processes underlying demyelination and remyelination [10-15]. Moreover, recent evidence suggests that LPC has an effect on pain. Topical LPC treatment was found to induce painful sensory phenomena (allodynia and hyperalgesia), focal demyelination and changes in the expression of some pain-related molecules [16]. These LPC-induced effects on pain transmission are similar to the reported effects of lysophosphatidic acid (LPA), since LPA_1 _receptor signaling initiates neuropathic pain and its underlying mechanisms, including demyelination, and alters the expression of pain-related molecules [17-19]. We speculate that centrally administered LPC may be converted to LPA by autotaxin (ATX) and cause LPA_1 _receptor activation. In accord with this proposal, we recently demonstrated that neuropathic pain induced by intrathecally (i.t.) administered LPC was abolished in mice deficient in LPA_1 _receptors (*Lpar1*^-/-^mice), and markedly attenuated in ATX gene heterozygous mutant (*atx*^+/-^) mice [20,21]. Furthermore we demonstrated that intense stimulation of spinal cord slices with pain transmitters or capsaicin, which is thought to release pain transmitters, caused a biosynthesis of LPC. This LPC was subsequently converted to LPA in the presence of recombinant ATX (rATX) [22]. These findings suggest that neuropathic pain induced by i.t. administered LPC occurs after its conversion to LPA and subsequent activation of LPA_1 _receptor signaling. The present study was an initial neurochemical examination of the processes underlying LPC-induced LPA production, and provides evidence of feed-forward LPA production through the LPA_3 _receptor.

## Methods

### Animals

Male C57BL/6J mice (Tagawa experimental animal laboratory, Japan), heterozygous mutant mice for the ATX gene (*atx*^+/-^) [23], and homozygous mutant mice for the LPA_1 _[24], LPA_2 _[25] and LPA_3 _[26] receptor genes (*Lpar1*^-/-^, *Lpar2*^-/- ^and *Lpar3*^-/-^), and their sibling wild-type (WT) mice from the same genetic background were used in this experiment, unless stated otherwise. The subjects weighed 20-24 g. They were kept in a room maintained at 21 ± 2°C, 55 ± 5% relative humidity and a 12 h light/dark cycle and had free access to a standard laboratory diet and tap water. The procedures were approved by the Nagasaki University Animal Care Committee, which complied with the fundamental guidelines for proper conduct of animal experiments and related activities in academic research institutions under the jurisdiction of the Ministry of Education, Culture, Sports, Science and Technology, Japan.

### Drugs

LPC (18:1), LPA (18:1) and sphingosine-1-phosphate (S1P) were purchased from Sigma (MO, USA). LPC and LPA were dissolved in artificial cerebrospinal fluid (aCSF: NaCl 125 mM, KCl 3.8 mM, KH_2_PO_4 _1.2 mM, NaHCO_3 _26 mM, glucose 10 mM) when they were used *in vivo*. In the *in vitro *experiments, LPA and S1P were dissolved in Dulbecco's Modified Eagle's Medium (DMEM) with 0.1% fatty acid-free bovine serum albumin (A-6003 Sigma-Aldrich, St. Louis, MO, USA).

### Recombinant ATX

In accord with previous experiments involving rat proteins [27], mouse cDNA for ATX was introduced into the baculovirus transfer vector pFASTBac-1 (Invitrogen, Carlsbad, CA, USA), and recombinant baculovirus was prepared according to the manufacturer's protocol. After purification using a baculovirus system and nickel column chromatography (HisTrap HP; Amersham Biosciences, Osaka, Japan), rATX was obtained from 1 L of culture supernatant of Sf9 insect cells infected with ATX recombinant baculovirus.

### Sample preparation from tissues

**Figure 1:**
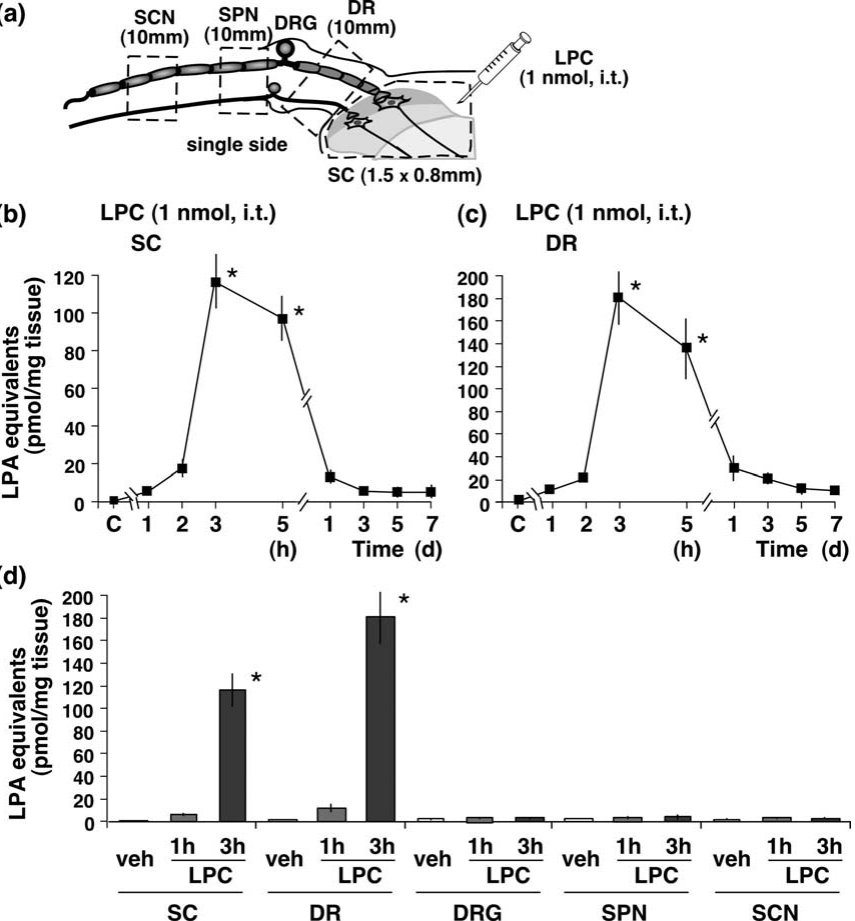
**Lysophosphatidylcholine (LPC) induces lysophosphatidic acid (LPA) production in the spinal dorsal horn and dorsal roots**. (a) The exact regions of removed samples, including dorsal horn of lumbar spinal cord (SC), dorsal roots (DR), dorsal root ganglions (DRG), spinal nerves (SPN) and sciatic nerves (SCN). (b and c) Quantification of LPA production at different time points after the LPC treatment. The capital letter "C" showed on the time course axis represents the vehicle group. (d) LPC-induced LPA production in different preparations from SC, DR, DRG, SPN and SCN at 1 and 3 h post-treatment. The measurement of LPA was carried out in triplicate of each sample. All data represent the mean ± SEM from three separate experiments. Rounding morphology was evaluated in at least 500 enhanced green fluorescence-positive cells. *p < 0.05 compared with the vehicle group.

At different time-points (see Results for details) after the injection of LPC or LPA, mice were anesthetized by pentobarbital (50 mg/kg, i.p.). The bilateral dorsal horn (Lamina I-V) of the lumbar (L4-6) spinal cord (SC), L4-6 dorsal roots (DR), L4-6 dorsal root ganglions (DRG), L4-6 spinal nerves (SPN) and L4-6 sciatic nerves (SCN) were then removed to enable the extraction of LPA, as shown in Fig. 1a. The average wet weights of isolated bilateral SC, DR, DRG, SPN and SCN in each mouse were 8, 4, 4, 4 and 5 mg, respectively. Following isolation, these tissue samples were put into polypropylene 1.5 mL tubes and homogenized by sonication in 300 μL serum-free DMEM solution for approximately 30 sec. To extract LPA from the homogenates using the solid-phase lipid extraction method, the sample was then slowly loaded onto Oasis HLB cartridges (Millipore, Tokyo, Japan), which had been pre-conditioned with 3 mL of methanol, followed by 3 mL of distilled water. The column was washed with 3 mL of distilled water then 1 mL of chloroform. LPA was then eluted with 600 μL methanol and dried up with N_2 _gas. The final sample was dissolved with 100 μL of DMEM solution and stored at -80°C until the biological assay.

### Biological titration method

B103 cells expressing LPA_1 _receptors and enhanced green fluorescence protein [B103 (+) cells] were used for the quantitative measurement of LPA, according to a method [22] modified from the earlier report [28]. The cells were maintained as monolayer cultures on tissue culture dishes in DMEM supplemented with a 10% heat-inactivated fetal bovine serum (Gibco, Carlsbad, CA, USA) that included penicillin and streptomycin (final concentration 100 units/mL). Cells were seeded at 2.5 × 10^4 ^cells/cm^2 ^onto 8-well slide glass coated with poly-L-lysine (Sigma, MO, USA; final concentration 100 mg/L) and collagen (BD Bioscience, San Jose, CA, USA; final concentration 5 μg/cm^2^). Next, they were cultured in DMEM containing 10% heat-inactivated fetal bovine serum at 37°C in a 5% CO_2 _atmosphere for 10 h. Cells were then cultured in serum-starved DMEM for 15 h, in accord with a previous study [22].

In the biological assay, either standard LPA or a diluted tissue sample was applied to B103 (+) cells. After incubation for 20 min at 37°C, the medium was replaced by 4% paraformaldehyde and incubated for another 60 min at 25°C. The slide glass was then cover-slipped with Fluoromount™ (DBS, Pleasanton, CA, USA) and examined under a fluorescence microscope (Keyence, Osaka, Japan). The percentage of cells that exhibited a rounded morphology was determined in at least 500 cells in each well.

### Preparation of spinal cord slices

Mice were anesthetized using pentobarbital (50 mg/kg, i.p.) with 1% xylocaine. The lumbar spinal cord was removed within 5 min and placed in ice-cold Krebs buffer (117.0 mM NaCl, 3.6 mM KCl, 1.2 mM NaH_2_PO_4_, 1.2 mM MgCl_2_, 25.0 mM NaHCO_3_, 5.0 mM CaCl_2 _and 11.0 mM glucose) containing EGTA (1 mM), and aerated with 95% O_2 _and 5% CO_2 _(carbogen) at pH 7.4. After the meninges were carefully removed, the rostral end of the lumbar spinal cord was glued and supported by a block of agar (1%). Transverse slices (500 μm) were then prepared in Krebs buffer, under a controlled temperature of 4°C and constant aeration with carbogen. Cutting was performed using a super microslicer ZERO (DSK, Kyoto, Japan) equipped with a ceramic knife. After sectioning, the slices were washed with Krebs buffer and serum-starved DMEM. Ten slices were then incubated in 100 μl of serum-starved DMEM in 96-well culture dishes at 37°C in 5% CO_2 _for 30 min. Just before drug treatment, 50 μl of medium was removed. 50 μl of each drug at double the final concentration was then added to each well. After incubation, the culture medium was collected and filtered using a nylon filter net (NRS-100; 13-XX, 100 μm pore; Nippon Rikagaku Kikai, Tokyo, Japan) to remove any fragments of spinal cord tissue. Half of each culture medium was used in the experiments evaluating the cell rounding effects.

### Nociceptive tests

In thermal paw withdrawal tests, nociception was measured as the latency to paw withdrawal evoked by exposure to a thermal stimulus [29,30]. Unanesthetized animals were placed in Plexiglas cages on top of a glass sheet and were allowed an adaptation period of 1 h. A thermal stimulator (IITC Inc., Woodland Hills, CA, USA) was positioned under the glass sheet and the focus of the projection bulb was aimed precisely at the middle of the plantar surface of the animal. A mirror attached to the stimulator permitted visualization of the plantar surface. A cut-off time of 20 sec was set in order to prevent tissue damage. The paw pressure test was performed, as described previously [30]. Mice were placed into a Plexiglas chamber on a 6 × 6 mm wire mesh grid floor and allowed to acclimatize for a period of 1 h. A mechanical stimulus was then delivered onto the middle of the plantar surface of the right hind-paw using a Transducer Indicator (Model 1601, IITC Inc., Woodland Hills, CA, USA). The pressure needed to induce a flexor response was defined as the pain threshold. All behavioral experiments were performed under double blind conditions.

### RT-PCR

The expression levels of LPA receptors in SC and DR were evaluated by reverse transcription polymerase chain reaction (RT-PCR), according to described method [31]. The L4-6 SC and DR were removed from naïve mice and lysed with TRIzol (Invitrogen, Carlsbad, CA, USA) for RNA preparation. Total RNA (1 μg/sample) was used for cDNA synthesis with PrimeScript^® ^RT reagent Kit (Takara, Otsu, Japan). A 1: 3 dilution series of the products was amplified by PCR. The cycling conditions for all primers were 3 min at 95°C, then 33 cycles of 30 s at 95°C, 30 s at 55°C and 2 min at 72°C. The glyceraldehyde-3-phosphate dehydrogenase (GAPDH) mRNA was used as a control. The PCR primer sequences were as follows: LPA_1_, 5'-ATCTTTGGCTATGTTCGCCA-3' (forward) and 5'-TTGCTGTGAACTCCAGCCA-3' (reverse); for LPA_2_, 5'-TGGCCTACCTCTTCCTCATGTTCCA-3' (forward) and 5'-GTGTCCAGCACACCACAAATGCC-3' (reverse); for LPA_3_, 3'-TTGCCTCTGCAACATCTCGG-3' (forward) and 5'-CATGACGGAGTTGAGCAGTG-3' (reverse); and for GAPDH, 5'-CAAGGTCATCCATGACAACTTTG-3' (forward) and 5'-GGCCATCCACAGTCTTCTGG-3' (reverse). Then, the PCR products were analyzed by 1.5% agarose gel electrophoresis.

### Statistical analysis

Statistical analysis was evaluated using the Student's t-test and Tukey's multiple comparison *post hoc *analysis following one-way ANOVA. The criterion of significance was set at *p *< 0.05. All results are expressed as mean ± SEM.

## Results

### LPC induces LPA production in the spinal dorsal horn and dorsal roots

For the measurement of LPA production, we adopted a biological titration method using LPA_1 _receptor-expressing B103 [B103 (+)] cells, according to the methods outlined in [28] and [22]. Using this method, we evaluated the percentages of cells showing a rounded morphology induced by the addition of LPA, examining at least 500 cells in each well. This measure was found to be specific to LPA, since 100 nM (equivalent: 10 pmol/100 μl) of both LPC [22] and S1P had no effect (Additional file 1, Fig. S1a). In addition, 100 nM LPA and extracts from samples produced no substantial effect on cell rounding in B103 cells without LPA_1 _receptor [B103 (-)] cells (Additional file 1; Fig. S1b). In B103 (+) cells, a linear equation of the induction of cell rounding activity was established for LPA from 0.15 to 5 pmol, after subtracting the basal cell rounding activity. Experiments were carried out in 100 μl wells. The equation was defined as y = 5.454x + 5.66 (R^2 ^= 0.991; x: log_10 _[LPA (pmol)]; y: cell rounding percentage; Additional file 1; Fig. S1c). In subsequent studies, LPA equivalents in the extracts from the dorsal horn of the spinal cord or dorsal roots were estimated using this equation based on linear LPA concentration-dependent responses.

The basal level of LPA-equivalents in the spinal dorsal horn (SC) of control mice was 0.66 pmol/mg tissue. The LPA levels were markedly increased when mice were i.t. injected with 1 nmol of LPC. The increase was maximal (117 pmol/mg tissue) at 3 h, slightly declined at 5 h, and had disappeared by day 1 (Fig. 1b). An increase in LPC-induced LPA was observed with a similar time-course in dorsal root (DR) preparations. The maximum level was 181 pmol/mg tissue (Fig. 1c), which was significantly higher than that in the dorsal horn. As the isolated SC (Lamina I-V) and DR (L4-6) weighed 8 and 4 mg respectively, the level of LPA production was approximately 936 pmol for SC and 724 pmol for DR at 3 h after treatment (117 pmol/mg tissue × 8 mg = 936 pmol; 181 pmol/mg tissue × 4 mg = 724 pmol). Therefore, the total synthesis of LPA is likely to have been at least 1.66 nmol (936 pmol + 724 pmol), which is higher than the original level of LPC at 1 nmol. A similarly high level of LPA was also observed at 5 h post-treatment. In contrast, no significant increase in LPA levels was observed in the preparations of DRG, SPN or SCN, as shown in Fig. 1d. In addition, there were no significant differences in the basal level of LPA equivalents among any of these preparations (Fig. 1d).

### ATX and LPA_3 _receptors are required in LPC-induced LPA production

**Figure 2:**
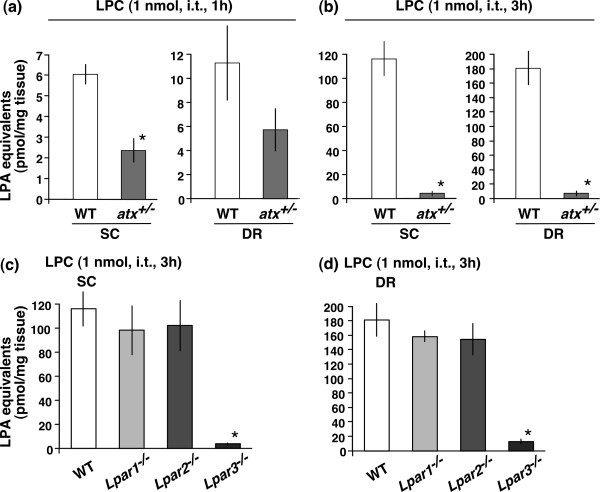
**ATX and LPA_3 _receptors are required for LPC-induced LPA production**. (a and b) Quantification of LPA production by LPC-treated tissue extraction from the SC and DR of wild-type (WT) mice and ATXgene heterozygous mutant (*atx*^+/-^) mice at 1 (*panel a*) and 3 h (*panel b*) post-treatment. (c and d) LPC-induced LPA production in the SC (*panel c*) and DR (*panel d*) from WT, *Lpar1*^-/-^, *Lpar2*^-/- ^and *Lpar3*^-/- ^mice at 3 h post-treatment. Three female *atx*^+/- ^mice were used in this experiment, but no significant difference was observed compared with other male mice. *p < 0.05 compared with the WT group. Other details are shown in the legend of Figure 1.

LPC-induced LPA production in the SC and DR was attenuated in *atx*^+/- ^mice at 1 h after LPC treatment, compared with WT mice (Fig. 2a). As expected, the LPA level in SC and DR of *atx*^+/- ^mice was decreased to 38 and 50% of control (WT) mice, respectively, suggesting that LPC is converted to LPA by the action of endogenous ATX. However, the level in WT mice was markedly increased to approximately 117 and 181 pmol/mg protein in the SC and DR respectively, as long as 3 h after the LPC treatment. Since the LPA level at 1 h was approximately 6 and 11 pmol/mg proteins in the SC and DR respectively, time-dependent changes were calculated as a 20-fold increase in the SC, and a 16-fold increase in the DR (Fig. 2b). Interestingly, the LPA levels in the SC and DR of *atx*^+/- ^mice at 3 h were both below 4% of WT mice (Fig. 2b), while LPC-induced LPA production in the SC and DR was completely absent in *Lpar3*^-/- ^mice, but not in *Lpar1*^-/- ^or *Lpar2*^-/- ^mice (Fig. 2c and 2d). On the other hand, three kinds of LPA_1_, LPA_2 _and LPA_3 _receptors were all expressed in SC and DR by RT-PCR, though the level of LPA_1 _transcripts was much higher than the others (Additional file 2; Fig. S2).

### LPA induces LPA production through LPA_3 _receptor in the spinal dorsal horn and dorsal roots

**Figure 3:**
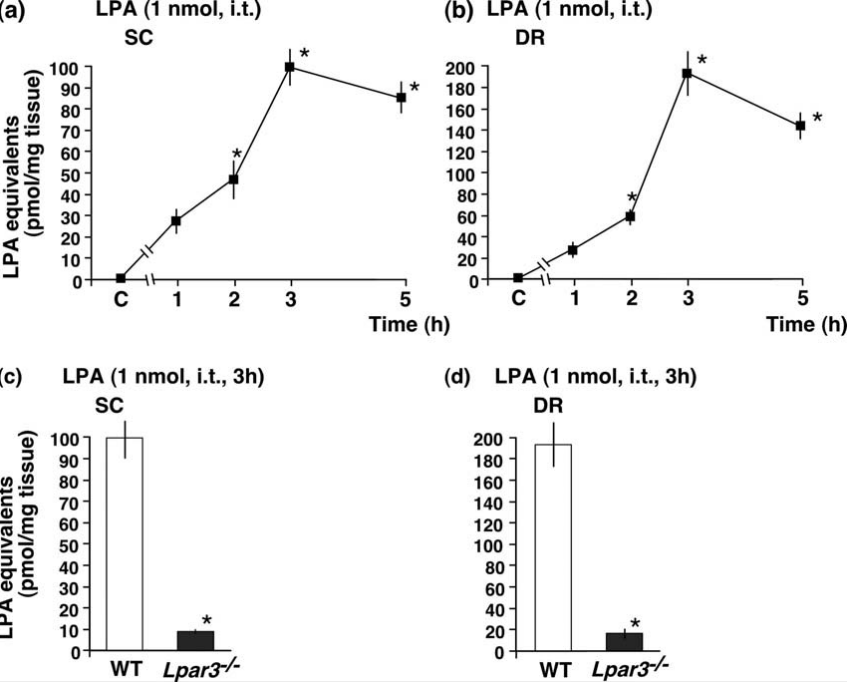
**LPA induces amplified LPA production through LPA_3 _receptor in spinal dorsal horn and dorsal roots**. (a and b) Time course of LPA production induced by LPA-treated tissue extraction from SC and DR. The capital letter "C" showed on the time course axis represents the vehicle group. (c and d) LPA_3 _receptor-dependent LPA production by LPA treatment. *p < 0.05 compared with the vehicle or WT group. Other details are shown in the legend of Figure 1.

When 1 nmol LPA was i.t. injected, the LPA level in the SC was increased in a time-dependent manner. A significant increase was observed at 2 h, with a maximum level at 3 h, followed by a slight decline at 5 h. The maximum level at 3 h (approximately 100 pmol/mg protein) was equivalent to that observed with LPC at 1 nmol (i.t.), as shown in Fig. 3a. Similar levels were observed in the DR (Fig. 3b). Calculations using the method described above suggest that the total level of LPA [100 pmol/mg tissue × 8 mg (SC) + 193 pmol/mg tissue × 4 mg (DR) = 1572 pmol] was higher than the LPA level at 1 nmol. LPA-induced LPA production was also entirely absent in *Lpar3*^-/- ^mice (Fig. 3c and 3d).

### *In vitro *evidence for LPA-induced LPA production through ATX and LPA_3 _receptors

**Figure 4:**
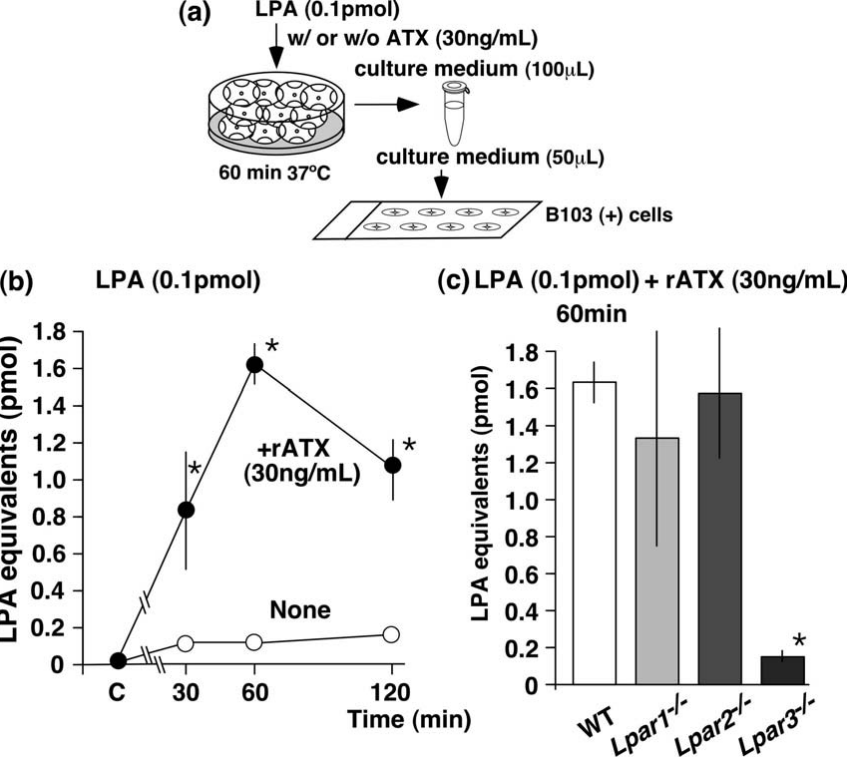
**LPA induces ATX-mediated LPA production through the LPA_3 _receptor *in vitro***. (a) Schematic representation of the method used. Details are described in the text. (b) Quantification of LPA production by LPA (0.1 pmol)-treated culture media with or without recombinant ATX (rATX, 30 ng/ml). The capital letter "C" showed in the time course axis represents the vehicle group. (c) LPA_3 _receptor-dependent LPA production by LPA and rATX. All data represent the mean ± SEM from three to seven separate experiments. *p < 0.05 compared with the vehicle or WT group. Other details are shown in the legend of Figure 1.

Ten transverse slices of the lumbar spinal cord were cut and washed using Krebs buffer several times to remove cerebrospinal fluids (CSF), which contain high levels of ATX [22,32]. These slices were incubated in serum-starved DMEM for 30 min at 37°C, followed by LPA application for the indicated time periods, with or without rATX. The supernatant culture medium was then collected from these cultured spinal cord slices. After filtration, the collected medium was applied to B103 (+) cells for 20 min at 37°C, and LPA activity was evaluated (Fig. 4a). In the presence of rATX (30 ng/mL), the addition of LPA at 0.1 pmol increased the LPA level in a time-dependent manner from 30 to 60 min, followed by a slight decline (Fig. 4b). However, no significant increase was observed in the absence of rATX. In addition, LPA-induced LPA production in the presence of rATX was significantly decreased in *Lpar3*^-/- ^mice, but not in *Lpar1*^-/- ^or *Lpar2*^-/- ^mice (Fig. 4c). As the ATX-dependent LPA production was detected in the supernatant of spinal cord slices in the present *in vitro *assay, it appears that this synthesis occurs in the extracellular space.

### LPA_1 _and LPA_3 _receptors are required in LPA-induced hyperalgesia and allodynia

**Figure 5:**
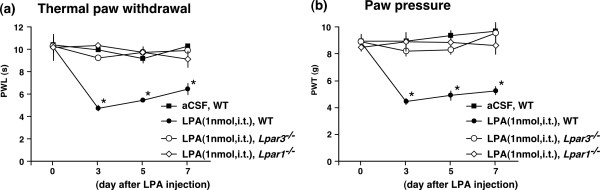
**LPA_1 _and LPA_3 _receptors are involved in LPA-induced neuropathic pain**. LPA (1 nmol) or aCSF was i.t. injected, and the thermal paw withdrawal test (*panel a*) and paw pressure test (*panel b*) were performed at days 3, 5 and 7 after injection. Results represent the threshold of latency (s) or pressure (g) to thermal (a) or mechanical (b) stimulus, respectively. All data represent the mean ± SEM from 3 mice. *p < 0.05 compared with the aCSF group.

LPA i.t. injection (1 nmol) in WT mice caused robust thermal hyperalgesia and mechanical allodynia for at least 7 days, while aCSF injection produced no change in thresholds (Fig. 5). These findings are consistent with our previous report [17]. LPA-induced hyperalgesia and allodynia were completely absent in *Lpar1*^-/- ^and *Lpar3*^-/- ^mice at days 3, 5, and 7 after injection (Fig. 5).

## Discussion

Here we demonstrated for the first time that i.t. injected LPC is not only converted to LPA through an action of endogenous ATX, but also causes a new synthesis of LPA *in vivo *in a feed-forward manner through the LPA_3 _receptor. Previous studies examining the involvement of endogenous ATX reported that it is a crucial enzyme for the conversion of LPC to LPA [21,33-36], and that high levels of ATX are expressed in CSF [22,32]. In this study, we used a biological titration assay, in which LPA_1 _receptor-mediated cell rounding activity was measured as a quantitative evaluation of LPA levels in processed extracts from tissue samples. In this assay, LPA concentration was detectable from 0.15 pmol (equivalent: 1.5 nM). This demonstrates the higher sensitivity of this assay compared with the enzymatic cycling method, another widely used method for LPA determination that is only suitable for concentrations over 100 nM LPA [37]. Furthermore, the addition of S1P to B103 (+) cells, and the addition of LPC or LPA to B103 (-) cells did not significantly increase cell rounding. Considering these findings together, the present biological assay method appears to be useful for the quantitation of low levels of LPA in extracts, as previously reported [22,28]. Because the addition of LPC to B103(+) cells did not cause cell rounding activity [22], the increase in LPA levels in the SC and DR after i.t. LPC injection is likely to be caused by the synthesis of new LPA. The lack of LPA production in the DRG, SPN or SCN may be simply related to the topological distance from the injection site of LPC. Since there are several subspecies of LPA [38,39], however, the advanced method for measurement of each species of LPA molecules utilizing mass spectrometry including highly efficient purification and condensation would be the next subject.

The LPA level in *atx*^+/- ^mice, which have been found to express only 50% of both the ATX levels and the lysophospholipase D (lyso-PLD) activity of WT mice [23], was just half that of the WT mice at 1 h following LPC injection. These findings suggest that the rapid production of LPA can be attributed to the ATX-mediated conversion of LPC to LPA. Although the experiment using the ATX inhibitor may support this hypothesis, commercially available ATX inhibitor has some affinities to LPA_1_, LPA_2 _and LPA_3 _receptors.

A second important issue is the time-dependent increase in LPA levels caused by injections of LPA as well as LPC. I.t. injection of LPA (1 nmol) caused a time-dependent increase in LPA levels in the SC and DR that lasted until 3 h post-treatment, followed by a slight decline in LPA levels at 5 h. This decline may be caused by the product-inhibition of ATX, since ATX activity is inhibited by high levels of LPA [40]. LPA-induced LPA production was also observed in the presence, but not in the absence of ATX in the *in vitro *experiment using spinal cord slices. LPA-induced production of LPA has also been previously found in an *in vitro *study using ovarian cancer cells. It was reported that phosphatidylcholine was first converted to phosphatidic acid (PA) by phospholipase D (PLD) followed by further hydrolysis to LPA by phospholipase A (PLA_2_) [41]. This finding of LPA production independent of ATX in ovarian cancer cells may suggest the existence of a different type of LPA synthetic pathway to that found in the central nervous system in the present study.

A similar level and time-course of LPA synthesis was observed following LPC injection (1 nmol, i.t.). The LPA synthesis induced by LPC in both the SC and DR was reduced to the basal level by 1 day and remained at this level for the succeeding days, possibly because of degradation and diffusion. It should be noted that LPA production induced by LPC markedly increased with time, until 3 h had passed. In contrast, the increase observed in *atx*^+/- ^mice was severely limited, suggesting that LPC-induced LPA production largely depends on the ATX action *in vivo*. This fact may be explained by the possibility that the LPA converted from LPC by ATX causes a new synthesis of LPC and this cycle occurs several times over 3 h.

Finally, it should be noted that LPA-induced *de novo *LPC (or converted LPA) synthesis in SC and DR was completely absent in *Lpar3*^-/- ^mice, but not in *Lpar1*^-/- ^and *Lpar2*^-/- ^mice, though LPA_1_, LPA_2 _and LPA_3 _receptors were all expressed in SC and DR. This finding was observed in both *in vivo *and *in vitro *experiments using spinal cord sections, and was subsequently supported by nociceptive tests showing that LPA-induced thermal hyperalgesia and mechanical allodynia were absent in *Lpar3*^-/- ^mice. This result is consistent with previous reports suggesting that LPA_2 _and LPA_3 _receptors might contribute to LPA-induced LPA production in ovarian cancer cells, because ovarian cancer cells express high levels of LPA_2 _and LPA_3 _receptors whereas normal ovarian epithelial cells express low levels of LPA_2 _and LPA_3 _receptors [41-43]. However, it is currently unclear which cell types in the spinal cord are involved in LPA production. It is particularly difficult to clarify whether the new synthesis of LPA occurs in specific neurons or in highly differentiated cell types. Alternatively, LPA synthesis may occur through neuron-glia interactions as well as in an autocrine manner. In the present study, we found that the LPA_3 _receptor is responsible for LPA synthesis. According to previous reports, the LPA_3 _gene in mice is expressed in astrocytes, but not microglias [44-46]. There are currently no reports detailing the expression of the LPA_3 _receptor in mouse neurons. An important aim of future research will be evaluating the role of LPA_3_-related molecular mechanisms of LPA-induced LPA production in single or co-cultured specific cell types.

The present findings can also be considered in relation to the mechanisms underlying neuropathic pain. In a series of studies, we have demonstrated that LPA_1 _receptor signaling initiates nerve injury- or LPA -induced (via i.t. injection) neuropathic pain and its underlying mechanisms. These mechanisms include up-regulation of the expression of the voltage-gated calcium channel α_2 _δ-1 subunit (Caα_2_δ-1) in the DRG, protein kinase Cγ (PKCγ) in the spinal dorsal horn and demyelination of dorsal root fibers as well as Aβ-fiber-mediated spinal reorganization [17-19,47,48]. Most recently, a pharmacological study has shown that LPA_1 _signaling within a 3 h timeframe can cause neuropathic pain [49]. As such, it is reasonable to speculate that the LPA_1 _and LPA_3 _receptors have differential key roles in the biological processes underlying the mechanisms of neuropathic pain and the amplification of LPA production, respectively.

## Conclusion

Our study provides the first demonstration that LPA can induce feed-forward LPA synthesis, a result that emerged in both *in vivo *and *in vitro *experiments. This feed-forward synthesis involves ATX-mediated conversion of LPC to LPA and LPA_3 _receptor-mediated LPC production. These mechanisms appear to be repeatedly active in a relatively short period within 3 to 5 h. This result is consistent with previous findings that LPA is a key molecule in the initiation of mechanisms underlying neuropathic pain.

## List of abbreviations

LPC: lysophosphatidylcholine; LPA: lysophosphatidic acid; ATX: autotaxin; *Lpar1*^-/-^, *Lpar2*^-/- ^and *Lpar3*^-/-^: LPA_1_, LPA_2 _and LPA_3 _receptor deficient mice; *atx*^+/-^: ATX gene heterozygous mutant mice; rATX: recombinant ATX; WT: wild-type mice; i.t.: intrathecal; S1P: sphingosine-1-phosphate; aCSF: artificial cerebrospinal fluid; DMEM: Dulbecco's Modified Eagle's Medium; SC: dorsal horn of lumbar spinal cord; DR: dorsal roots; DRG: dorsal root ganglions; SPN: spinal nerves; SCN: sciatic nerves; B103 (+): LPA_1 _receptor-expressing B103 cells; B103 (-): LPA_1 _receptor-lacking B103 cells; CSF: cerebrospinal fluids; RT-PCR: reverse transcription polymerase chain reaction; GAPDH: glyceraldehyde-3-phosphate dehydrogenase; lyso-PLD: lysophospholipase D; PA: phosphatidic acid; PLD: phospholipase D; PLA_2_: phospholipase A; Caα_2_δ-1: voltage-gated calcium channel α_2_δ-1 subunit; PKCγ: protein kinase Cγ

## Competing interests

The authors declare that they have no competing interests.

## Authors' contributions

LM is responsible for performance of overall experiments and writing the manuscript. H Uchida and JN participated in the RT-PCR study and LPA measurements, respectively. MI participated in the LPA measurement using spinal cord slices. JC generated LPA_1_, LPA_2 _and LPA_3 _receptor deficient mice. JA generated recombinant ATX and ATX gene heterozygous mutant mice. HU is responsible for the experimental design and writing the manuscript. All authors read and approved the final manuscript.

## Supplementary Material

Additional file 1 **Specificity of LPA measurements using a measure of cell rounding activity in B103 cells**. (a) Cell rounding activity of added LPA or S1P to B103 (+) cells. (b) Cell rounding activity of added LPA or tissue extracts to B103 (-) cells. (c) Linearity (y = 5.454x + 5.66, R^2 ^= 0.991) of cell rounding-inducing activity for LPA between 0.15 and 5 pmol in B103 (+) cells, after subtracting the basal activity. All data represent the mean ± SEM from three to four separate experiments. *p < 0.05 compared with the vehicle group. Other details are shown in the legend of Figure 1. Click here for file

Additional file 2 **Gene expression analysis for LPA_1_, LPA_2 _and LPA_3 _receptors in the spinal cord dorsal horn and dorsal root**. Expression of LPA_1_, LPA_2 _and LPA_3 _receptors in mouse spinal cord dorsal horn (SC) and dorsal root (DR) by RT-PCR. Click here for file
