# Supplementary figures and images for: Lysophosphatidic acid-3 receptor-mediated feed-forward production of lysophosphatidic acid: an initiator of nerve injury-induced neuropathic pain
Source: Mol Pain. 2009 Nov 13;5:64. doi: 10.1186/1744-8069-5-64 (PMC2780384; doi:10.1186/1744-8069-5-64)

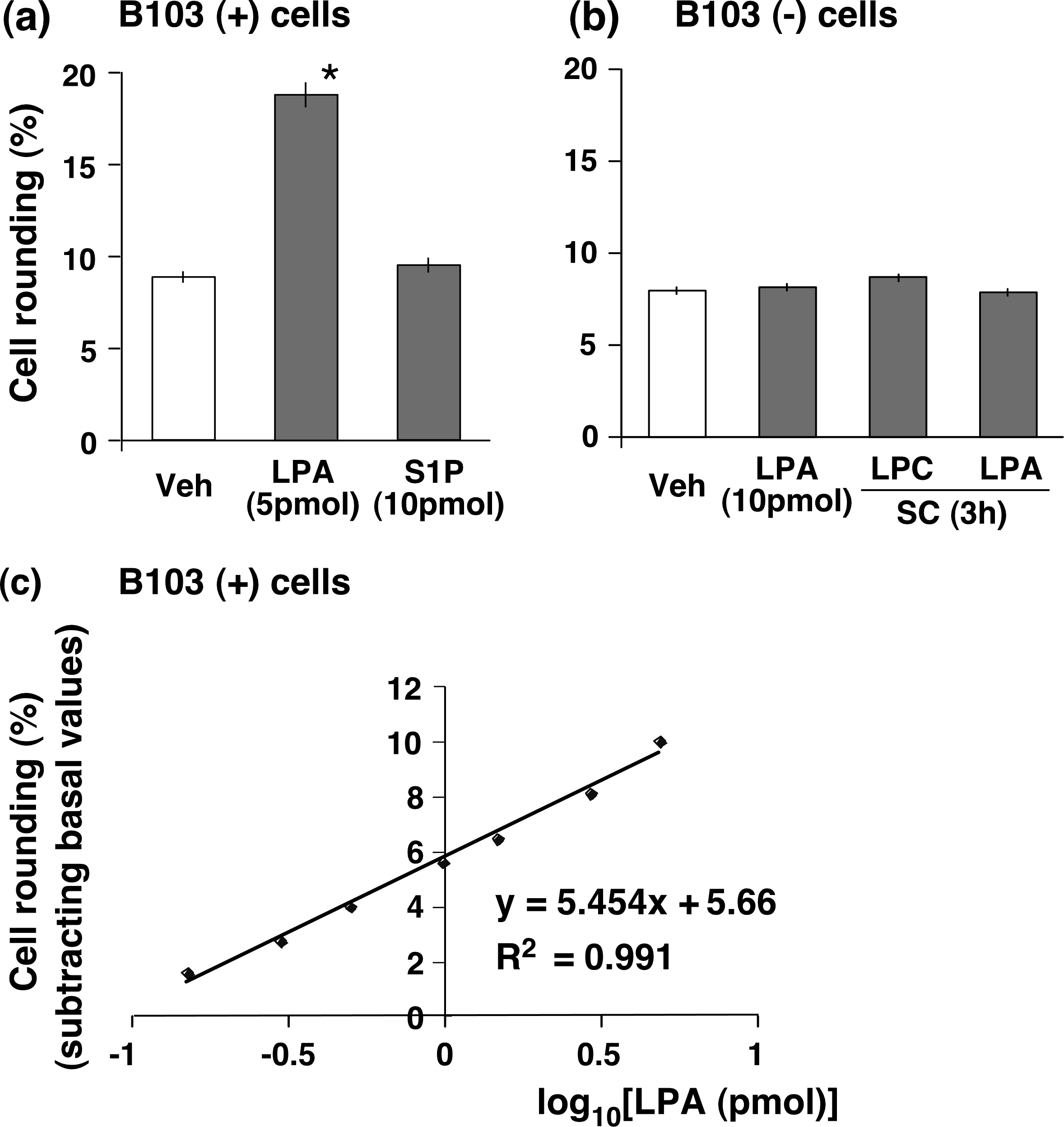

Supplement: Additional file 1 — Specificity of LPA measurements using a measure of cell rounding activity in B103 cells. (a) Cell rounding activity of added LPA or S1P to B103 (+) cells. (b) Cell rounding activity of added LPA or tissue extracts to B103 (-) cells. (c) Linearity (y = 5.454x + 5.66, R2 = 0.991) of cell rounding-inducing activity for LPA between 0.15 and 5 pmol in B103 (+) cells, after subtracting the basal activity. All data represent the mean ± SEM from three to four separate experiments. *p < 0.05 compared with the vehicle group. Other details are shown in the legend of Figure 1. [file 1744-8069-5-64-S1.tiff]

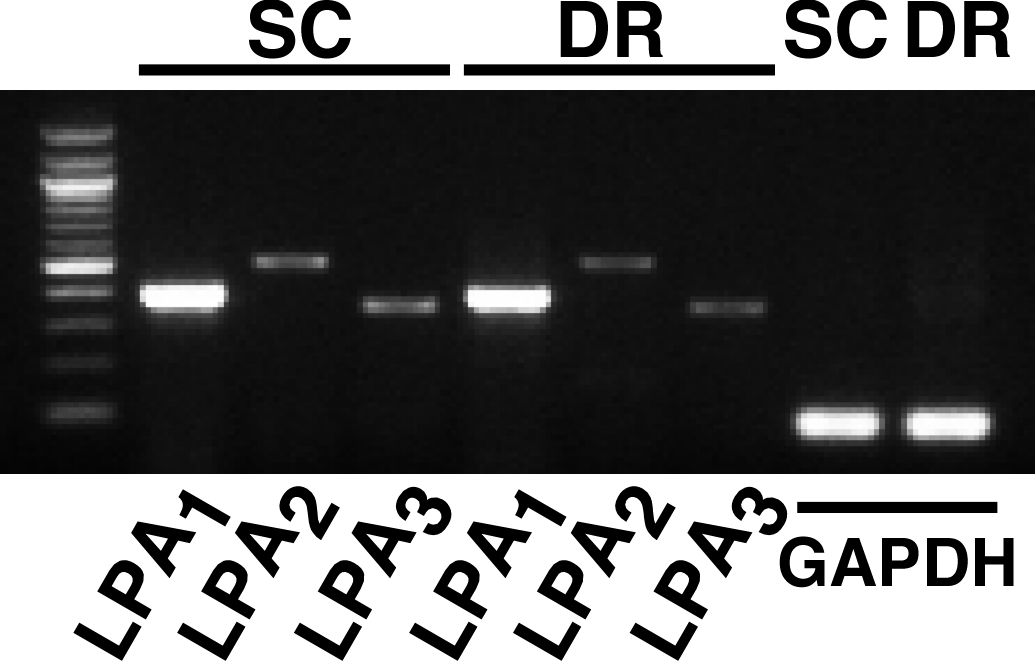

Supplement: Additional file 2 — Gene expression analysis for LPA1, LPA2 and LPA3 receptors in the spinal cord dorsal horn and dorsal root. Expression of LPA1, LPA2 and LPA3 receptors in mouse spinal cord dorsal horn (SC) and dorsal root (DR) by RT-PCR. [file 1744-8069-5-64-S2.tiff]
